# ATG3 Is Important for the Chorion Ultrastructure During Oogenesis in the Insect Vector *Rhodnius prolixus*

**DOI:** 10.3389/fphys.2021.638026

**Published:** 2021-02-03

**Authors:** Anna Santos, Isabela Ramos

**Affiliations:** ^1^Laboratório de Bioquímica de Insetos, Instituto de Bioquímica Médica Leopoldo de Meis, Universidade Federal do Rio de Janeiro, Paraná, Brazil; ^2^Instituto Nacional de Ciência e Tecnologia em Entomologia Molecular – INCT-EM/CNPq, Rio de Janeiro, Brazil

**Keywords:** ATG3, choriogenesis, *Rhodnius prolixus*, vector biology, autophagy related 3

## Abstract

In insects, the last stage of the oogenesis is the choriogenesis, a process where the multiple layers of the chorion are synthesized, secreted, and deposited in the surface of the oocytes by the follicle cells. The chorion is an extracellular matrix that serves as a highly specialized protective shield for the embryo, being crucial to impair water loss and to allow gas exchange throughout development. The E2-like enzyme ATG3 (autophagy related gene 3) is known for its canonical function in the autophagy pathway, in the conjugation of the ubiquitin-like ATG8/LC3 to the membranes of autophagosomes. Although the ATGs were originally described and annotated as genes related to autophagy, additional functions have been attributed to various of these genes. Here, we found that *Rhodnius prolixus* ATG3 is highly expressed in the ovaries of the adult vitellogenic females. Parental RNAi depletion of ATG3 resulted in a 15% decrease in the oviposition rates of depleted females and in the generation of unviable eggs. ATG3-depleted eggs are small and present one specific phenotype of altered chorion ultrastructure, observed by high resolution scanning electron microscopy. The amounts of the major chorion proteins Rp30, Rp45, Rp100, and Rp200 were decreased in the ATG3-depleted chorions, as well as the readings for dityrosine cross-linking and sulfur, detected by fluorescence emission under ultraviolet excitation and X-ray elemental detection and mapping. Altogether, we found that ATG3 is important for the proper chorion biogenesis and, therefore, crucial for this vector reproduction.

## Introduction

The ability of insects to produce a large number of eggs in a short period of time is one of the characteristics that contributes to their remarkable adaptive success in inhabiting many different habitats and acting as vectors of human diseases. Exploring the specific mechanisms of egg formation and embryo development should be of great importance to further understand the reproductive biology of various vectors and to elaborate new strategies for vector population control (Pereira et al., 2020). This is especially important for the neglected, vector borne tropical diseases, which are endemic to developing countries such as dengue fever and Chagas Disease^1^.

In oviparous animals, including insects, the female germline cells enter meiosis, while they differentiate into a highly specialized cell designed to support embryo development after fertilization. The cytoplasm of a mature oocyte is characterized by the presence of accumulated maternal mRNAs, ribosomes and mitochondria, as well as set of endocytic-originated vesicles named yolk organelles that usually occupy most of the oocyte volume (Kerkut and Gilbert, 1985; Kunkel and Nordin, 1985; Alberts et al., 2002). In the latest stage of oogenesis, the follicle cells (a single layered tissue of professional secretory cells that surround the oocytes during oogenesis) go through the major metabolic challenge of rapidly synthesizing and secreting the chorion layers (Huebner and Anderson, 1972; Wu et al., 2008). After choriogenesis the mature oocyte is ready to be fertilized and laid in the environment. In species that colonize land, the chorion layers allow gas exchange and serve as a shielding barrier to impair water loss during embryogenesis (Kerkut and Gilbert, 1985; Bomfim et al., 2017). The process of chorion formation and its complex ultrastructure represent a remarkable model for *in vivo* studies of biogenesis and assembly of the network of macromolecules in an extracellular matrix. In *Drosophila*, studies about choriogenesis focus mostly on the programed gene-specific transcriptional activation of chorion genes (Tootle et al., 2011; Velentzas et al., 2018) and on the biochemical characterization of chorion proteins, mainly by mass spectrometry (Fakhouri et al., 2006). In *Rhodnius prolixus*, the chorion ultrastructure and permeability properties were previously explored (Beament, 1948; Dias et al., 2013; Bomfim et al., 2017; Bomfim and Ramos, 2020) and the identification of the specific chorion proteins Rp30 and Rp45, the latter associated to an antifungal activity, were also described (Bouts et al., 2007).

Autophagy is an intracellular degradation pathway in which target organelles or complexes are sequestered by an expanded double membrane vesicle generating an organelle named autophagosome. After fusion of the autophagosome with the lysosome, the degradation products of autophagy are reutilized by the cell as a source of building blocks for the synthesis of new macromolecules (Klionsky et al., 2016). Autophagy is a well-conserved mechanism throughout evolution in eukaryotic cells and it is carried out by a set of conserved autophagy related genes (ATGs) that can be found from yeast to mammals. The autophagy pathway includes two essential ubiquitin-like conjugation systems (Mizushima et al., 1998; Ichimura et al., 2000; Ohsumi, 2001). In one of them, the ubiquitin-like molecule ATG12 is activated by the E1-like enzyme ATG7, transferred to the E2-like conjugating enzyme ATG10, and in the end attached to ATG5 (Mizushima et al., 1998; Tanida et al., 1999; Nemoto et al., 2003). In the other, ATG7 and the E2-like enzyme ATG3 conjugate the ubiquitin-like ATG8/LC3 to the lipid phosphatidylethanolamine in the autophagosomes (Tanida et al., 1999, 2002; Ichimura et al., 2000; Ohsumi, 2001). Although the ATGs have been used for the study of autophagy since their discovery in 1993 in *Saccharomyces cerevisiae* (Tsukada and Ohsumi, 1993), additional non-autophagic functions have been assigned to these genes (Subramani and Malhotra, 2013; Galluzzi and Green, 2019). It was recently identified that ATG3 also regulates late endosomal trafficking, cell proliferation, LC3-associated phagocytosis and exosome release (Sanjuan et al., 2007; Martinez et al., 2015; Murrow and Debnath, 2015, 2018; O’Sullivan et al., 2016).

Here, we found that *R. prolixus* ATG3 is highly expressed in the ovaries of vitellogenic females. RNAi depletion of ATG3 resulted in a small decrease in the oviposition rates and the generation of unviable eggs. ATG3-depleted eggs were small and presented a specific phenotype of altered chorion ultrastructure as seen by high resolution scanning electron microscopy. Although the major chorion proteins Rp30, Rp45, Rp100, and Rp200 were detected in the ATG3-depleted chorions, their levels were reduced. The readings for cross-linking of chorion proteins, as seen by the reduced fluorescence emission under ultraviolet excitation, which indicates lower levels of dityrosine crosslinking were also reduced. Finally, ATG3-depleted chorions were also deficient in sulfur, a marker for protein presence and potential cysteine protein cross-linking, as detected by X-ray elemental detection and mapping. Altogether, we found that ATG3 is important for the proper chorion biogenesis and, therefore, crucial for this vector reproduction.

## Materials and Methods

### Ethics Statement

All animal care and experimental protocols were approved by guidelines of the institutional care and use committee (Committee for Evaluation of Animal Use for Research from the Federal University of Rio de Janeiro, CEUA-UFRJ #01200.001568/2013-87, order number 155/13), under the regulation of the national council of animal experimentation control (CONCEA). Technicians dedicated to the animal facility conducted all aspects related to animal care under strict guidelines to ensure careful and consistent animal handling.

### Insects

Insects were maintained at a 28 ± 2°C controlled temperature and relative humidity of 70–80%. Mated females are fed for the first time (as adult insects) in live-rabbit blood 14 to 21 days after the 5th instar nymph to adult ecdysis. After the first blood feeding, all adult insects in our insectarium are fed every 21 days. For all experiments, mated females of the second or third blood feeding were used. All animal care and experimental protocols were approved by the guidelines previously described in the ethics statement.

### Gene Identification

The sequence of the *R. prolixus* ATG3 transcript was identified as one single isoform of the gene *RpATG3* (RPRC008742) corresponding to the transcript RPRC008742-RA in the *R. prolixus* genome assembly (RproC3.4) available at Vector Base^2^. The identification was accessed by similarity to the *Drosophila melanogaster* ATG3 sequence (Gene ID 40044) using tBlastn under default settings. Conserved domains were detected using PFAM. Alignments of the ATG3 protein sequence to the ATG3 sequences from different species were performed using Clustal Omega.

### Extraction of RNA and cDNA Synthesis

All organs were dissected 7 days after blood meal and homogenized in Trizol reagent (Invitrogen) for total RNA extraction. Reverse transcription reaction was carried out using the High Capacity cDNA Reverse Transcription Kit (Applied Biosystems), using 1 μg of total RNA after RNase-free DNase I (Invitrogen) treatment, all according to the manufacturer’s protocol.

### PCR/RT-qPCR

Specific primers for *R. prolixus* ATG3 sequence were designed to amplify a 203 bp fragment in a PCR using the following cycling parameters: 5 min at 95°C, followed by 35 cycles of 30 s at 95°C, 30 s at 52°C and 30 s at 72°C and a final extension of 15 min at 72°C. Amplifications were observed in 2% agarose gels. Quantitative PCR (RT-qPCR) was performed in a StepOne Real-Time PCR System (Applied Biosystems) using SYBR Green PCR Master Mix (Applied Biosystems) under the following conditions: 10 min at 95°C, followed by 40 cycles of 30 s at 95°C and 30 s at 60°C. RT-qPCR amplification was performed using the specific primers listed on Supplementary Table S1. The relative expressions were calculated using the delta C_t_ (cycle threshold) obtained using the reference gene 18S (RPRC017412) and calculated 2^–*dCt*^. For all RT-qPCRs the samples for each biological replicate were dissected from a pool of three insects.

### RNAi Depletion

dsRNA was synthesized by MEGAScript RNAi Kit (Ambion Inc.) using primers for *R. prolixus ATG3* specific gene amplification with the T7 promoter sequence designed to target a region of 647 bp. Unfed adult females were injected between the second and third thoracic segments using a 10 μL Hamilton syringe with 1 μg dsRNA (in a volume of 1 μL) and fed 2 days later. Seven days after the blood feeding, the knockdown efficiency was confirmed by RT-qPCR. A fragment of 808 bp of the Escherichia *coli MalE* gene (Gene ID: 948538) included in the control plasmid LITMUS 28iMal obtained from the HiScribe RNAi Transcription kit (New England BioLabs) was amplified by PCR using a T7 promoter-specific primer, targeting the opposing T7 promoters of the vector. The cycling conditions were: 10 min at 95°C, followed by 35 cycles of 30 s at 95°C, 30 s at 52°C and 60 s at 72°C and a final extension of 15 min at 72°C. The amplified fragment was used as a template for the synthesis of the control dsRNA (dsMal). Adult females injected with dsRNAs were fed and transferred to individual vials.

### Evaluation of Survival and Egg Laying

All groups injected with dsRNAs (10 insects per batch, 3–4 batches per treatment) were kept separately in transparent plastic jars. Mortality was recorded daily. Egg laying was recorded weekly. Additional measurements are described below. Three experiments were performed, each of them containing at least 10 insects per treatment.

### Chorion Protein Extraction and SDS-PAGE

A total of 0 to 72 h eggs were collected and their chorions were carefully washed in 0.01 M Tris/HCl pH 8.4 several times. Following, the chorions from five control and ATG3-depleted eggs were treated as previously described by Bouts et al. (2007). Briefly, the chorions were homogenized in 8 M urea, 360 mM Tris/HCl (pH 8.4) and 30 mM dithiothreitol using a glass/teflon potter Elvehjem homogenizer and solubilized at room temperature for 10 min. The samples were centrifuged at 12,000 *g* for 10 min, and the supernatant was collected and stored at −20°C for further use. The small precipitate obtained during centrifugation was discarded. 10 μl of the urea-extracted proteins were applied to a 10% SDS-PAGE and stained with silver nitrate (Merril et al., 1981). Densitometry was performed using ImageJ.

### Field Emission Scanning Electron Microscopy (SEM)

Freshly laid eggs were carefully collected and fixed by immersion in 2.5% glutaraldehyde (Grade I) and 4% freshly prepared formaldehyde in 0.1 M cacodylate buffer, pH 7.3. Samples were washed in cacodylate buffer, dehydrated in an ethanol series, critical point dried and coated with a thin layer of gold. Models were observed in a FEI Quanta 250 field emission scanning electron microscope operating at 15 kV.

### Dityrosine Chorion Fluorescence

Eggs were photographed using a Zeiss Axiozoom V.16 stereomicroscope using a DAPI filter set for dityrosine fluorescence detection, as previously described by Dias et al. (2013). Comparison of fluorescence levels was performed using the same objectives and exposure times. For fluorescence quantification, images of eight eggs each from control and depleted females were analyzed with ImageJ software. To calculate the approximate chorion fluorescence (CF), we used the following formula: CF = integrated density − (area of selected egg × mean fluorescence of background readings).

### Energy Dispersive X-Ray Microanalysis and Elemental Mapping

For X-ray microanalysis, the eggs were collected at times ranging from 0 to 24 h after oviposition and were directly examined in a Hitachi TM4000Plus scanning electron microscope operating at 15 kV. For spectra and elemental mapping, X-rays were collected for 100 s using a Silicon drift detector (SDD) on a 0 to 10 KeV energy range with a resolution of 158 eV.

## Results

### *R. prolixus* ATG3 Is Highly Expressed in the Ovaries and Oocytes During Vitellogenesis

**FIGURE 1:**
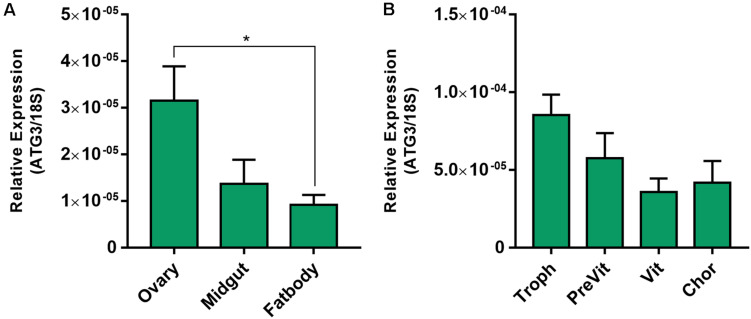
ATG3 is highly expressed in the ovaries and developing follicles of vitellogenic females. **(A)** RT-qPCR showing the ATG3 mRNA quantification in the different organs of vitellogenic females dissected 7 days after the blood meal. **(B)** RT-qPCR showing the ATG3 mRNA quantification in the different components of the ovariole: Tropharium, pre-vitellogenic oocytes, vitellogenic oocytes and chorionated oocytes. The relative expression was quantified using the ΔCT method. Graphs show mean ± SEM (*n* = 4). *p < 0.05, one-way ANOVA.

*Rhodnius prolixus* ATG3 encodes a predicted protein with 309 amino acid residues and 76/64% similarity/identity with the human ATG3. All the expected ATG3 conserved domains (PF03986, Autophagy_N; PF03987, Autophagy_act_C; PF10381, Autophagy_C) were detected (Supplementary Figure S1). Using quantitative PCR (RT-qPCR) we found that the ovary of vitellogenic females express an average of 3× more ATG3 than the midgut and fat body (Figure 1A). Within the ovary, ATG3 mRNA was detected in the tropharium (structure where the germ cell cluster and the nurse cells are located) and in all stages of the developing follicles (pre-vitellogenic, vitellogenic, and chorionated) (Figure 1B).

### RNAi Depletion of ATG3 Results in Decreased Oviposition and Altered Chorion Ultrastructure

**FIGURE 2:**
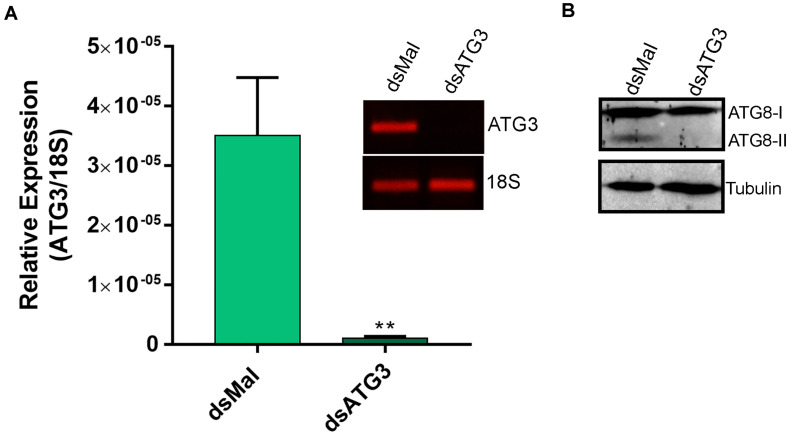
ATG3 is depleted in the ovary of vitellogenic females. **(A)** RT-qPCR showing the levels of ATG3 mRNA in control (dsMal) and ATG3-depleted (dsATG3) ovaries dissected 7 days after the blood meal. Graph shows mean ± SEM (*n* = 5). ***p < 0.001, Student’s *t* test. Inset: 25-cycle PCR fragments showing the amplification of ATG3 and 18S in control and depleted ovaries 7 days after the blood meal. **(B)** ATG8/LC3 Immunoblotting on dsMal and dsATG3 ovary samples. ATG8-I, non-lipidated ATG8; ATG8-II, ATG8 conjugated to the phosphatidylethanolamine.

**FIGURE 3:**
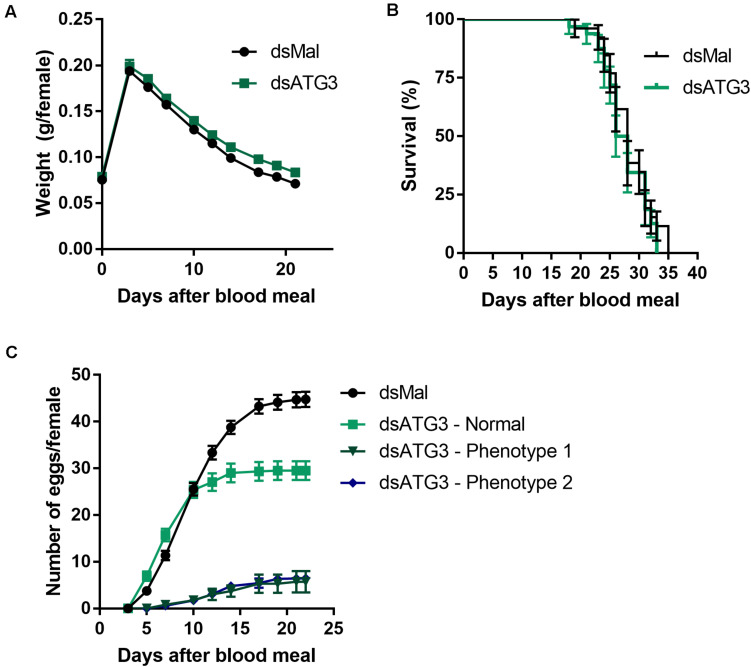
Depletion of ATG3 does not affect digestion and longevity of the vitellogenic females. **(A)** Control and ATG3-depleted females were weighted for different days after the blood meal. **(B)** Survival rates of control and ATG3-depleted females. **(C)** Oviposition rates of control and depleted females over the gonotrophic cycle. All graphs show mean ± SEM (*n* = 30).

**FIGURE 4:**
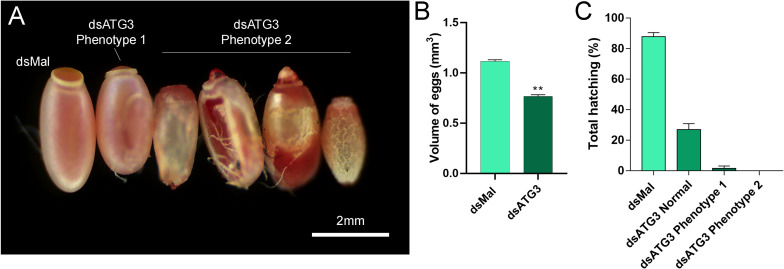
Parental depletion of ATG3 results in small, chorion-defective, and unviable eggs. **(A)** Representative images of control and ATG3-depleted eggs. The two types of observed phenotypes are indicated. **(B)** Approximated calculated volume of control and ATG3-depleted eggs (*n* = 12). **(C)** Hatching rates of the embryos from control and ATG3-depleted females (*n* = 24). Graphs show mean ± SEM. *p < 0.05, **p < 0.01, one-way ANOVA.

To investigate the role of ATG3 in the ovary, we synthesized one specific double-stranded RNA designed to target the sequence of *R. prolixus* ATG3 and injected it directly into the females hemocoel, 2 days before the blood meal. RT-qPCR showed that the ATG3 knockdown was efficient, with an average of 95% mRNA silencing in the ovaries of vitellogenic females. The bacterial *MalE* gene was used as a control dsRNA (dsMal) (Figure 2A). Because ATG3 is one of the enzymes responsible for the conjugation of ATG8/LC3 to the autophagosome membrane, we performed immunoblottings to address the ATG8 lipidation status in control and depleted ovaries. We found that depletion of ATG3 resulted in decreased levels of ATG8/LC3 lipidation (ATG8-II), as expected (Figure 2B), suggesting that autophagosomes were not properly formed in the ovaries of ATG3-depleted females. The depletion of ATG3 did not result in alterations in the main physiological characteristics of the adult female such as digestion, as measured by the weight of the insects (Figure 3A), and longevity (median survival of 28 days for control females and 27 days for depleted females, *p* > 0.05) (Figure 3B). To investigate the role of ATG3 during oogenesis, we quantified the oviposition in control and ATG3-depleted females and maintained the collected eggs under control conditions to ensure embryo development. Our results show a 18% decrease in the total number of eggs laid by ATG3-depleted females (Figure 3C). In addition to the reduced oviposition, we observed that the ATG3-depleted females laid eggs presenting two main types of abnormal phenotypes, shown in Figure 4A. Per female, an average of four eggs (10% of the total number of eggs) were smaller than controls but showed no apparent defects in the chorion structure (phenotype 1), and 5 eggs (12% of the total number of eggs) were smaller than controls and showed evident defects in the chorion structure (phenotype 2) (Figures 4A,B). In total, we observed a 70% decrease in the embryo viability of ATG3-depleted females when compared to control females. All the eggs that presented one of the phenotypes described above were unviable and only 30% of the eggs that presented an apparent normal morphology were viable (Figure 4C).

**FIGURE 5:**
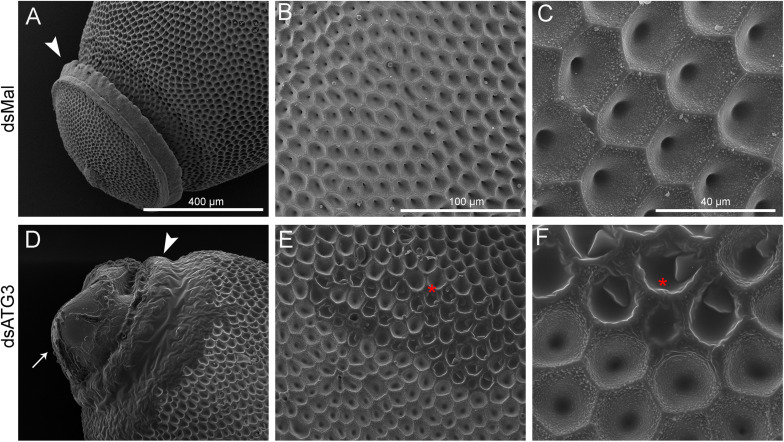
Depletion of ATG3 results in defective chorion ultrastructure. Freshly laid eggs were processed for scanning electron microscopy (SEM). **(A–C)** SEM images of the chorion of control (dsMal) eggs in increasing magnifications. **(A)** Detail of the anterior pole of the egg. Arrowhead: operculum. **(B)** Transitional magnification image of the exochorion surface. **(C)** High magnification image of the exochorion surface showing the typical hexagonal patterns of the chorion in *R. prolixus*. **(D–F)** SEM images of the chorion of ATG3-depleted eggs in increasing magnifications. **(D)** Detail of the anterior pole of the ATG3-depleted egg sowing the altered structure of the operculum (arrowhead), and the leaking of the egg internal contents from the defective operculum (arrow). **(E)** Transitional magnification image of the exochorion surface of depleted eggs. **(C)** High magnification image of the exochorion surface showing the typical hexagonal patterns of the chorion in *R. prolixus* and the altered ultrastructure regions (asterisk). All images are representative of five eggs laid by at least five control and ATG3-depleted females (*n* = 5).

To further examine the ATG3 chorion defects (phenotype 2), control and ATG3-depleted eggs were processed for high resolution SEM and the ultrastructure of the external surface of the chorion was observed. We found that ATG3-depleted eggs presented major alterations in the structure of the operculum at the anterior pole of the egg (Figures 5A and D, arrowheads) (Tuft, 1950). Also, as shown in Figures 5A and D, the malformations in the chorion of the ATG3-depleted eggs result in leaking of internal contents out of the operculum (Figure 5D, arrow), and the embryo to dry out within a few hours after oviposition (not shown). As for the rest of the egg surface, control eggs are covered by a stiff chorion marked by the presence of irregular pentagonal or hexagonal depressions, which are demarcated by the spaces where the follicle cells were placed during oogenesis. In contrast ATG3-depleted eggs present regions where this general outline of the chorion structure is strongly altered (Figures 5B–F, red asterisks).

### The Altered Chorion Ultrastructure Is Due to Altered Protein Levels and Reduced Protein-Crosslinking

**FIGURE 6:**
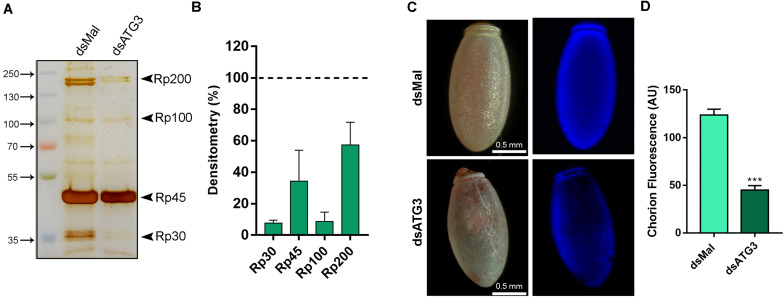
ATG3-depleted eggs present reduced levels of all the major chorion proteins and lower levels of dityrosine crosslinking. **(A)** 10% SDS-PAGE of the urea-extracted proteins from control and ATG3-depleted chorions, collected 0–24 h after oviposition. Arrowheads show Rp30, Rp45, Rp100, and Rp200. **(B)** Densitometry measurements of the chorion proteins indicated in **(A)** was performed using Image J. Graph shows mean ± SEM (*n* = 4). **(C)** Representative images of the dityrosine fluorescence in control and ATG3-depleted egg chorions. The fluorescence images were taken using a filter set for intrinsic dityrosine fluorescence. **(D)** Total chorion fluorescence was quantified using ImageJ software. Graph shows mean ± SEM (*n* = 10). ***p < 0.0001, Student’s *t* test. *AU*, arbitrary units.

**FIGURE 7:**
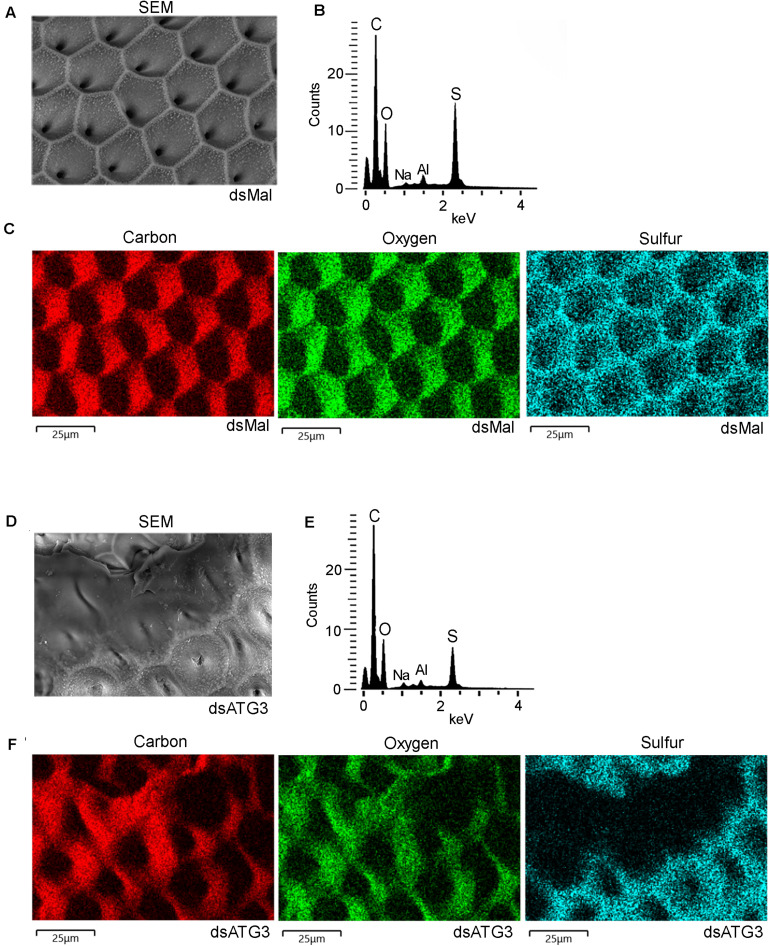
ATG3-depleted chorions present lower levels of sulfur, a marker of protein cross-linking. Control and ATG3-depleted chorions were analyzed by electron probe X-ray microanalysis. **(A)** Scanning electron microscopy image of the chorion from whole and unfixed control eggs. **(B)** X-ray spectrum corresponding to image in panel A. Aluminum (Al) peak in the spectrum came from the stub. **(C)** Elemental mapping of the chorion image shown in **(A)** reveals the co-localization of carbon (C), oxygen (O), sodium (Na) and sulfur (S). **(D)** Scanning electron microscopy image of the chorion from whole and unfixed ATG3-depleted egg. **(E)** X-ray spectrum corresponding to image in panel D. Aluminum (Al) peak in the spectrum came from the stub. **(F)** Elemental mapping of the chorion shown in **(D)** reveals sulfur is absent from the regions where the chorion shows the most severe abnormal ultrastructure. The images are representative of X-ray detections from three control and ATG3-depleted eggs. Bars: 25 μm.

To further investigate the nature of the defects in the chorion ultrastructure in ATG3-depleted eggs, we performed urea extractions of the main chorion proteins from control and ATG3-depleted insects, as previously described by Bouts et al. (2007). We found that ATG3-depleted chorions possess reduced levels of all the major chorion proteins detected by SDS-PAGE (Figures 6A,B). Rp30 and Rp100 are 90% decreased in ATG3-depleted eggs, whereas Rp45 and Rp200 are reduced only by approximately 50% (Figure 6B). Additionally, it is known that the hardening and waterproofing properties of the chorion in *R. prolixus* involve protein cross-linking through peroxidase-mediated oxidation of tyrosine residues (Li et al., 1996; Dias et al., 2013). To investigate the cross-linking properties in the chorion defective eggs, we measured the level of fluorescence of the ATG3-depleted chorions under ultraviolet excitation, a property of dityrosine formed by oxidative protein cross-linking (Neff et al., 2000; Andersen and Roepstorff, 2005). We found that the ATG3 defective chorions showed an average of 60% decrease in fluorescence emission under ultraviolet excitation (Figures 6C,D). Furthermore, X-ray microanalysis showed that the chorion possesses significant amounts of carbon, oxygen, and sulfur. The latter is a commonly used element-marker for the presence of proteins (Figures 7A,B). Elemental mapping of the image shown in Figure 7A, showed that these elements are evenly distributed over the chorion surface (Figures 7A–C). For the ATG3-depleted eggs, the content of sulfur in the chorion was generally reduced (Figures 7D,E), and the elemental mapping of the image in Figure 7D, showed that sulfur is mostly absent in the regions of the chorion that are present in the most altered ultrastructure (Figures 7D–F), indicating that the ATG3 abnormal ultrastructure is likely the result of both reduced protein levels and crosslinking.

## Discussion

In 2005, the WHO created the department of the neglected tropical diseases (NTDs), recognizing their importance and with the objective of managing their incidence. *R. prolixus* is a vector of Chagas disease, an important NTD in endemic regions of Latin American countries. Currently 7 million people are estimated to be infected by Chagas, and vector control is still the most useful method to prevent this illness (Nunes-da-fonseca et al., 2017; WHO, 2019). Here, we found that ATG3, a known component of the autophagy machinery, is essential for the chorion biogenesis and embryo viability in *R. prolixus*. We consider these findings significant because they provide knowledge on the biology of egg production of an important vector. Exploring and identifying the molecular machinery essential for egg formation is of crucial importance to further understand the reproductive biology of various vectors and might contribute to the development of new strategies for vector population control and the prevention of vector borne diseases such as Chagas Disease.

The participation of the autophagy machinery in different contexts of oogenesis and embryo development has been observed before in different models. The clearance of maternal mRNAs during the maternal to zygotic transition and the degradation of paternal mitochondria after fertilization were associated to an increase in the autophagic flux in mice and *Caenorhabditis elegans*, respectively (Al Rawi et al., 2011; Yamamoto et al., 2014). In Drosophila, different ATG mutants present varied phenotypes of impaired embryogenesis (McPhee and Baehrecke, 2010; Kuhn et al., 2015). In *R. prolixus*, members of the autophagy machinery, such as ATG6/Beclin1, ATG8/LC3, and ATG1/ULK1 are also highly expressed in the ovary and oocytes throughout vitellogenesis, and, like ATG3, their knockdown result in various oogenesis and impaired embryo phenotypes (Vieira et al., 2018; Bomfim and Ramos, 2020; Pereira et al., 2020).

The process of chorion formation represents a remarkable model system for studying *in vivo* the biogenesis of complex extracellular matrix architectures. In *Drosophila*, studies about choriogenesis focus mostly on the programed gene-specific transcriptional activation of chorion genes (Tootle et al., 2011; Velentzas et al., 2018), and on the biochemical characterization of chorion proteins by mass spectrometry (Fakhouri et al., 2006). The fact that LC3-lipidation was reduced in ATG3-depleted samples indicates that the autophagic canonical function of ATG3 was indeed altered in the oocytes. Thus, it is probable that, at least at some level, an autophagy impairment contributed to the altered chorion secretion/deposition ATG3 phenotype. However, because ATG3 has been implicated in various other cellular functions, such as endosomal trafficking, phagocytosis, cell proliferation, and exosome release (Murrow and Debnath, 2015, 2018), it is difficult to interpret the contribution of all those potential functions to the compromised secretion/exocytosis of the chorion proteins. Furthermore, although we tested *in silico* for predicted off targets using our dsRNA fragment against the *R. prolixus* transcriptome database and found no significate results, it is not possible to rule out potential off target effects of RNAi silencing. Despite the fact that the chorion biogenesis is essential for reproduction in oviparous animals, the machinery and signals encompassing the chorion proteins synthesis, sorting, and secretion by the follicle cells, are still largely unexplored. In *R. prolixus*, transcriptome analysis demonstrated that the follicle cells are highly committed to transcription, translation, protein turnover and vesicular traffic, as those functions accounted for more than two-thirds of all transcripts identified. Measurements of the expression of six transcripts involved in the unfolded protein response (UPR), a signaling network that coordinates the recovery of ER function in conditions of protein-folding stress, showed their expression to be increased during early stages of oogenesis and repressed at late stages, especially in the choriogenic follicles (Medeiros et al., 2011). Thus, it seems reasonable to assume that any molecular perturbation that somehow affect the follicle cells biosynthetic secretory pathway will result in some type of altered chorion structure. In fact, in previous work, our group has shown that parental RNAi depletion of ULK1/ATG1 in *R. prolixus* also resulted in oocytes with abnormal chorion ultrastructure. Unlike ATG3, the lipidation of LC3 was not affected, and it was shown that the ATG1/ULK1 depleted follicle cells displayed expanded rough ER membranes as well as increased expression of the ER chaperone BiP3, both indicatives of ER-stress. Plus, the knockdown of SEC16A, a known partner of the non-canonical ULK1/ATG1 function in the ER exit sites, phenocopied the depletion of ULK1/ATG1 (Bomfim and Ramos, 2020).

The stable biding of proteins through intermolecular covalent bonds is commonly referred to as protein cross-linking. It generates new macromolecular assemblies with different physicochemical properties and functions. The formation of specific disulfide bonds occurs as the protein folds in the ER where the linking of proper cysteines is coordinated by the action of a system of ER chaperones. For the maturation of extracellular proteins, correct cysteine crosslinking is essential to stabilize the protein as well to bind them to other proteins that will form the matrix network (Wilkinson and Gilbert, 2004; Heck et al., 2013). In this context, our findings that not all of the control chorion proteins are detected in the depleted samples, and that sulfur is decreased in the chorion layers of ATG3-depleted insects, indicates that specific proteins were not properly delivered to the chorion, either because they were not properly synthesized or folded or because they were not correctly secreted by the follicle cells. In addition, the mechanical rigidity and waterproofing properties of the chorion in *R. prolixus* has been attributed to protein cross-linking by peroxidases (Beament, 1946; Li et al., 1996; Konstandi et al., 2005; Li and Li, 2006; Dias et al., 2013) and the fact that we detected reduced dityrosine fluorescence signals provides additional evidence that the protein networks that are likely to form in the chorion layers were not properly formed in ATG3-depleted insects.

Most interestingly, the findings that ATG3 also participates in exosome release (Murrow and Debnath, 2015, 2018) raises the speculation that exosomes might be a part of the general chorion biogenesis. It has been shown that late endosomes can fuse with the plasma membrane leading to secretion of intralumenal vesicles as exosomes (Théry et al., 2002). Because the chorion is a highly specialized extracellular matrix, it is possible that some of the components are delivered to the eggshell packed in extracellular vesicles, such as exosomes. Experiments focusing on the follicle cells ultrastructure and *in vitro* secretion patterns, including the investigation of extracellular vesicles, are currently under investigation in our laboratory.

## Data Availability Statement

The original contributions presented in the study are included in the article/Supplementary Material, further inquiries can be directed to the corresponding author/s.

## Ethics Statement

The animal study was reviewed and approved by all animal care and experimental protocols were approved by guidelines of the institutional care and use committee (Committee for Evaluation of Animal Use for Research from the Federal University of Rio de Janeiro, CEUA-UFRJ #01200.001568/2013-87, order number 155/13), under the regulation of the national council of animal experimentation control (CONCEA).

## Author Contributions

AS designed and conducted the experiments and revised the manuscript. IR designed the experiments and wrote the manuscript. Both authors contributed to the article and approved the submitted version.

## Conflict of Interest

The authors declare that the research was conducted in the absence of any commercial or financial relationships that could be construed as a potential conflict of interest.

## Footnotes

1 www.who.int/neglected_diseases/en/

2 https://www.vectorbase.org/

## Supplementary Material

Supplementary Figure 1 *R. prolixus* ATG3 sequence. **(A)** Representation of ATG3 protein conserved domains in different species. Sequence information was obtained from Vector Base (https://www.vectorbase.org/). Conserved domains were obtained from the NCBI Conserved Domains Database. PF03986 (Autophagy_N); PF03987 (Autophagy_act_C); PF10381 (Autophagy_C). **(B)** Matrix of similarity and identity of ATG3 protein sequences from different species (SIAS Server). Reference sequences: *Rp, Rhodnius prolixus*; HsAtg3, *Homo sapiens* (Gene ID: 64422); DmAtg3, *Drosophila melanogaster* (Gene ID: 40044); BmAtg3, *Bombyx mori* (Gene ID: 100216351); ScAtg3, *Saccharomyces cerevisiae* (Gene ID: 855741).

Supplementary Table 1 Primers sequences. All sequences were obtained from *Vector Base* (https://www.vectorbase.org/) and primers were synthesized by Macrogen or IDT technologies. The T7 sequence is underlined.
