# Supplementary figures and images for: ATG3 Is Important for the Chorion Ultrastructure During Oogenesis in the Insect Vector Rhodnius prolixus
Source: Front Physiol. 2021 Feb 3;12:638026. doi: 10.3389/fphys.2021.638026 (PMC7888535; doi:10.3389/fphys.2021.638026)

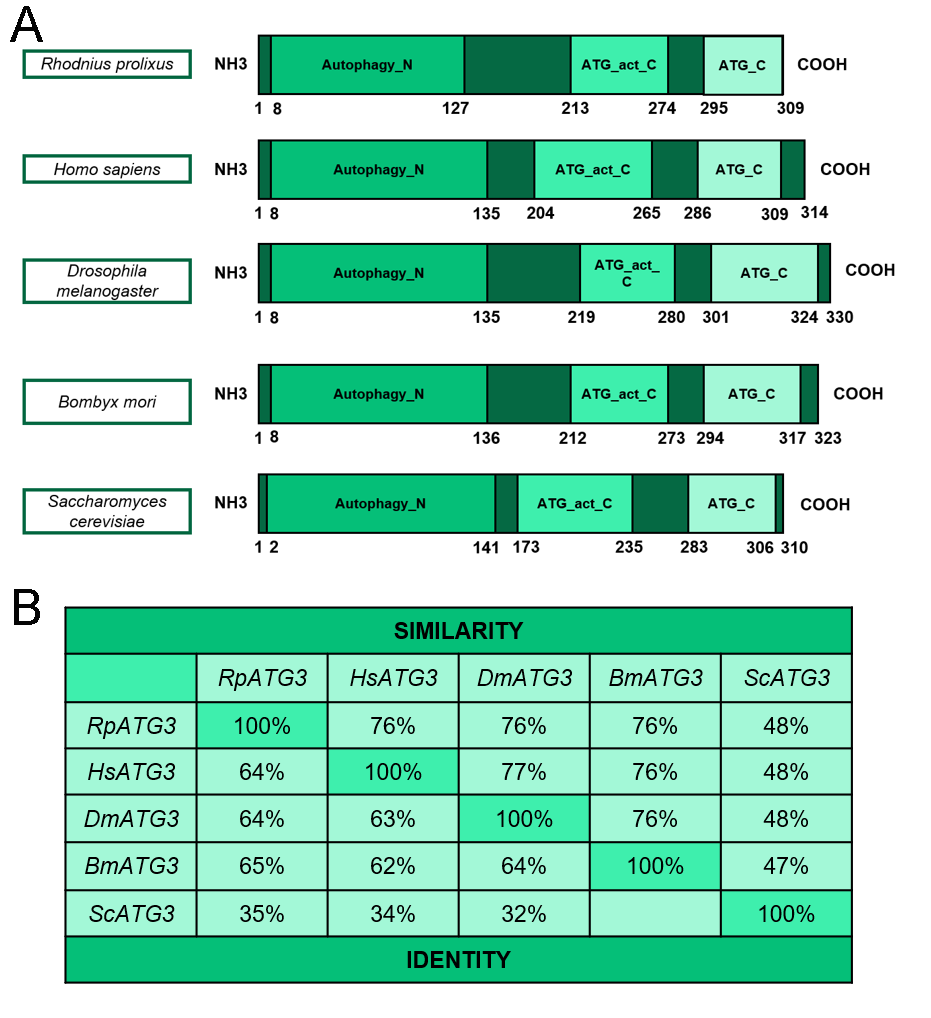

Supplement: Supplementary Figure 1 — R. prolixus ATG3 sequence. (A) Representation of ATG3 protein conserved domains in different species. Sequence information was obtained from Vector Base (https://www.vectorbase.org/). Conserved domains were obtained from the NCBI Conserved Domains Database. PF03986 (Autophagy_N); PF03987 (Autophagy_act_C); PF10381 (Autophagy_C). (B) Matrix of similarity and identity of ATG3 protein sequences from different species (SIAS Server). Reference sequences: Rp, Rhodnius prolixus; HsAtg3, Homo sapiens (Gene ID: 64422); DmAtg3, Drosophila melanogaster (Gene ID: 40044); BmAtg3, Bombyx mori (Gene ID: 100216351); ScAtg3, Saccharomyces cerevisiae (Gene ID: 855741). [file Image_1.tif]
