# Supplementary material for: ATG3 Is Important for the Chorion Ultrastructure During Oogenesis in the Insect Vector Rhodnius prolixus
Source: Front Physiol. 2021 Feb 3;12:638026. doi: 10.3389/fphys.2021.638026 (PMC7888535; doi:10.3389/fphys.2021.638026)
Supplement: Supplementary Table 1 — Primers sequences. All sequences were obtained from Vector Base (https://www.vectorbase.org/) and primers were synthesized by Macrogen or IDT technologies. The T7 sequence is underlined. [file Table_1.DOCX]

**Table S1: Primers Sequences**

| **Primer** | **Efficiency** | **Forward** | **Reverse** |
| --- | --- | --- | --- |
| *RpATG3*  *(RT-qPCR)* | 129% | 3’-CCAGAAGAATTTGTTGCTGCG-5’ | 3’-CGATTGATAGACCCCGACGATCC-5’ |
| *Rp18S*  *(RT-qPCR)* | 100.4% (Majerowicz et al., 2011) | 5’-TCGGCCAACAAAAGTACACA-3’ | 5’-TGTCGGTGTAACTGGCATGT-3’ |
| *RpATG3*  *(dsRNA)* | - | 5’-TAATACGACTCACTATAGGGTACTCCTTGTACTCGTCGCTGCA-3’ | 5’-TAATACGACTCACTATAGGGTACTGTGGCGGAGAACTTGGAGT -3’ |

All sequences were obtained from *Vector Base* (<https://www.vectorbase.org/>) and primers were synthesized by Macrogen or IDT technologies. The T7 sequence is underlined.
